# Verbal Working Memory Is Related to the Acquisition of Cross-Linguistic Phonological Regularities

**DOI:** 10.3389/fpsyg.2017.01487

**Published:** 2017-09-12

**Authors:** Evelyn Bosma, Wilbert Heeringa, Eric Hoekstra, Arjen Versloot, Elma Blom

**Affiliations:** ^1^Fryske Akademy Leeuwarden, Netherlands; ^2^Amsterdam Center for Language and Communication, University of Amsterdam Amsterdam, Netherlands; ^3^Leiden University Centre for Linguistics, Leiden University Leiden, Netherlands; ^4^Department of Modern Foreign Languages and Cultures, University of Amsterdam Amsterdam, Netherlands; ^5^Special Education Cognitive and Motor Disabilities, Department of Education and Pedagogy, Utrecht University Utrecht, Netherlands

**Keywords:** bilingualism, cognates, verbal working memory, cross-linguistic phonological regularities, minority language

## Abstract

Closely related languages share cross-linguistic phonological regularities, such as Frisian *-âld* [ͻ:t] and Dutch *-oud* [ʱut], as in the cognate pairs *kâld* [kͻ:t] – *koud* [kʱut] ‘cold’ and *wâld* [wͻ:t] – *woud* [wʱut] ‘forest’. Within Bybee’s (1995, 2001, 2008, 2010) network model, these regularities are, just like grammatical rules within a language, generalizations that emerge from schemas of phonologically and semantically related words. Previous research has shown that verbal working memory is related to the acquisition of grammar, but not vocabulary. This suggests that verbal working memory supports the acquisition of linguistic regularities. In order to test this hypothesis we investigated whether verbal working memory is also related to the acquisition of cross-linguistic phonological regularities. For three consecutive years, 5- to 8-year-old Frisian-Dutch bilingual children (*n* = 120) were tested annually on verbal working memory and a Frisian receptive vocabulary task that comprised four cognate categories: (1) identical cognates, (2) non-identical cognates that either do or (3) do not exhibit a phonological regularity between Frisian and Dutch, and (4) non-cognates. The results showed that verbal working memory had a significantly stronger effect on cognate category (2) than on the other three cognate categories. This suggests that verbal working memory is related to the acquisition of cross-linguistic phonological regularities. More generally, it confirms the hypothesis that verbal working memory plays a role in the acquisition of linguistic regularities.

## Introduction

Closely related languages such as Frisian and Dutch share cross-linguistic phonological regularities (Sjölin, 1976; Rys, 2009; Taeldeman, 2013). These regularities connect a fixed sequence of phonemes in one language to another fixed sequence of phonemes in the other language. An example of such a regularity is Frisian *-âld* [ͻ:t] and Dutch *-oud* [ʱut], as in the cognate pairs *kâld* [kͻ:t] – *koud* [kʱut] ‘cold’ and *wâld* [wͻ:t] – *woud* [wʱut] ‘forest’. However, not all cognate pairs follow a cross-linguistic regularity. For example, it is not the case that Frisian *a-* [a] as in *amer* [amər] always corresponds to Dutch *e-* [𝜀] as in *emmer* [𝜀mər] ‘bucket’. It is thought that bilingual speakers make use of cross-linguistic phonological regularities to relate the vocabulary of one language to the other and to quickly switch between languages (Sjölin, 1976; Rys, 2009; Taeldeman, 2013). However, as far as we know, there is no psycholinguistic evidence for this claim. Recent research, though, suggests that cross-linguistic phonological regularities do have a mental reality, as children seem to start using them as they grow older (Bosma et al., 2016).

In the present study, we investigated whether the acquisition of cross-linguistic phonological regularities is related to verbal working memory. This could not only give us more insight into the acquisition of these regularities themselves. As we will explain, it may also shed more light on the mechanisms that support language acquisition in general. In what follows, we will first describe our previous study (Bosma et al., 2016) in more detail, followed by a description of how the acquisition of cross-linguistic phonological regularities could be explained within Bybee’s (1995, 2001, 2008, 2010) usage-based network model. It was not our intention to test this model or to make theoretical statements. Rather, we used the model as a framework to describe and interpret regularities within the lexicon in a comprehensible way. Within the network model, applied to a bilingual learning context, phonological regularities across languages are similar to grammatical rules within a language. As the acquisition of grammar, but not vocabulary is supported by verbal working memory (Gottardo et al., 1996; McDonald, 2008; Engel de Abreu and Gathercole, 2012; Verhagen and Leseman, 2016), this suggests that verbal working memory supports the acquisition of linguistic regularities. If this is the case, then we would expect verbal working memory to be related to the acquisition of cognates with a cross-linguistic phonological regularity, but not to the acquisition of other types of cognates and non-cognates.

In a longitudinal study with three consecutive annual measurements, Bosma et al. (2016) tested 5- to 8-year-old Frisian-Dutch bilingual children on a Frisian receptive vocabulary task that comprised four cognate categories: (1) identical cognates, (2) non-identical cognates with a simple cross-linguistic phonological regularity (3) non-identical cognates without or with a more complex cross-linguistic phonological regularity, and (4) non-cognates. The results showed a gradual cognate facilitation effect for children with a low intensity of exposure to Frisian at home: the higher the degree of cross-language similarity, the better their performance. Furthermore, over time, the children with a low intensity of exposure to Frisian at home improved the most on non-identical cognates with a cross-linguistic phonological regularity. In the first and second year of the study, their performance on this type of cognates was comparable to their performance on non-identical cognates without such a regularity, whereas in the third year of the study, it was similar to their performance on identical cognates. This suggests that as they grow older, children become better at recognizing regularities between the Frisian and Dutch phonological systems.

The graduality of the cognate facilitation effect shows that a word in the input co-activates semantically and phonologically similar words in the other language depending on their degree of similarity. In fact, the spreading of activation in the bilingual lexicon is probably no different from the spreading of activation in the monolingual lexicon (Costa et al., 2005), which has also been shown to depend on the degree of phonological and semantic similarity between words (Gonnerman et al., 2007). This spreading of lexical activation as a function of similarity is the basis of Bybee’s (1995, 2001, 2008, 2010) network model, which proposes that the lexicon is a complex network of linguistic items in which phonologically and semantically related words are stored as spatially proximate. In this model, it is argued that similarity-based categorization and analogy are two of the domain-general mechanisms that support language acquisition. As speakers categorize linguistic items for storage, so-called schemas arise. These are organizational patterns in the lexicon that capture phonological and semantic generalizations about linguistic items. For example, English past tense verbs with the allomorph /d/ are stored together because they have the same final consonant and share past-tense meaning. The connections between these past tense forms lead to the identification of the suffix. When a speaker creates novel items based on analogy to this schema, the past tense suffix becomes productive. In contrast to what is traditionally thought of as grammar, the generalizations that arise from schemas in the lexicon do not necessarily have a cognitive representation that is independent of the individual linguistic items that together form the schema. This means that there is no separate storage of the rule. Within Bybee’s network model, grammar is not seen as a system that is separate from the lexicon [as in Pinker’s (1991) dual-processing model or Ullman’s (2004) declarative/procedural model], but rather as the structure that arises from the complex network of phonological and semantic relations within the lexicon.

As similarity-based activation of lexical items occurs both within (Gonnerman et al., 2007) and across languages (Dijkstra et al., 2010; Bosma et al., 2016), it can be assumed that phonologically and semantically similar words are stored closely together, regardless of whether they belong to the same or to a different language. Thus, the network model is not only able to account for regularities within a language, but also for regularities across languages. This suggests that cross-linguistic phonological regularities resemble grammatical rules, as they can both be thought of as generalizations that arise from schemas of phonologically and semantically related words.

Previous research has shown that grammar acquisition is related to verbal working memory (Gottardo et al., 1996; McDonald, 2008; Engel de Abreu and Gathercole, 2012; Verhagen and Leseman, 2016). The precise cognitive architecture of the verbal working memory system is still under debate, but although different researchers work with different definitions (for an overview, see Cowan, 2016), most views support that it is used for both the temporary storage, also referred to as verbal short-term memory, and the processing of verbal information. Following Baddeley and Hitch (1974) and Baddeley (1986), verbal short-term is thus considered to be part of the larger verbal working memory system. Verbal short-term memory has been shown to play a role in children’s first (L1) (Gathercole et al., 1992; Gathercole et al., 1997; Engel de Abreu and Gathercole, 2012; Verhagen and Leseman, 2016) and second language (L2) vocabulary acquisition (Cheung, 1996; Masoura and Gathercole, 2005; Engel de Abreu and Gathercole, 2012; Verhagen and Leseman, 2016) as well as in children’s L1 (Montgomery, 1995) and L2 grammar acquisition (French and O’Brien, 2008; Verhagen et al., 2015; Verhagen and Leseman, 2016). The processing component of verbal working memory is also argued to be important for children’s L1 (Gottardo et al., 1996; Engel de Abreu and Gathercole, 2012; Verhagen and Leseman, 2016) and L2 grammar acquisition (McDonald, 2008; Engel de Abreu and Gathercole, 2012; Verhagen and Leseman, 2016), as has been shown by studies involving receptive grammar (Engel de Abreu and Gathercole, 2012), sentence repetition (Verhagen and Leseman, 2016), grammaticality judgment (Gottardo et al., 1996; McDonald, 2008) and inflectional morphology (Verhagen and Leseman, 2016). However, no relationship has been found between verbal working memory and vocabulary acquisition (Engel de Abreu and Gathercole, 2012; Verhagen and Leseman, 2016). This suggests that verbal short-term memory and verbal working memory are differentially associated with language learning. As both vocabulary and grammar are related to verbal short-term memory, it is argued that the storage component of verbal working memory is important for the development of stable phonological representations in long-term memory (Baddeley et al., 1998). After all, children can only transfer words and multiword units to long-term memory after they have first stored them in short-term memory (Speidel, 1993).

The observation that verbal working memory is related to the acquisition of grammar, but not vocabulary suggests that verbal working memory is important for the processing of linguistic regularities. In terms of Bybee’s network model, this suggests that it plays a role in the formation of linguistic schemas through categorization and/or their productive use through analogy, a view that is supported by the finding that verbal working memory also plays a role in the categorization of non-linguistic items (Lewandowsky, 2011; Lewandowsky et al., 2012) and in non-linguistic analogical reasoning (Waltz et al., 2000).

In the current study, we investigated the hypothesis that verbal working memory is related to the acquisition of linguistic regularities. Although previous studies did not find a relationship between verbal working memory and the acquisition of vocabulary (Engel de Abreu and Gathercole, 2012; Verhagen and Leseman, 2016), we expected to find this relationship when the words follow a particular pattern. To this end, we investigated children’s vocabulary acquisition in a bilingual context with two closely related languages that share cross-linguistic phonological regularities. We hypothesized that verbal working memory would support the acquisition of cognates that follow a cross-linguistic phonological regularity, but not the acquisition of other types of cognates and non-cognates. In order to answer this question, we used the longitudinal data from the 5- to 8-year-old children in our previous cognate study (Bosma et al., 2016) and investigated associations with verbal working memory, thereby controlling for verbal short-term memory (Engel de Abreu and Gathercole, 2012), SES (Rice and Hoffman, 2015), exposure (Pearson et al., 1997), non-verbal IQ (Rice and Hoffman, 2015) and age, which have previously been shown to be related to vocabulary learning.

## Materials and Methods

### Participants

Participants were recruited by contacting primary schools in the countryside of the Dutch province of Fryslân. A total of 122 children from 14 different schools took part in the first year of our study (61 girls and 61 boys). Two children dropped out after the first wave of data collection, leaving 120 children in the second and third year of the study (61 girls and 59 boys). They were 5- or 6-years-old at time 1, 6- or 7-years-old at time 2 and 7- or 8-years-old at time 3. **Table 1** provides an overview of participants’ age, non-verbal IQ scores, socioeconomic status (SES) and intensity of exposure to Frisian at home. Non-verbal IQ was measured with the subsets Matrices and Recognition of the *Wechsler Non-verbal Scale of Ability* (WNV; Wechsler and Naglieri, 2006). Information about SES and intensity of exposure to Frisian at home were obtained through a parental questionnaire, based on the *Questionnaire for Parents of Bilingual Children* (PaBiQ) (Cost Action ISO804, 2011; Tuller, 2015). SES was calculated as the mean educational level of the father and the mother of the child, which was measured on a 1 to 9 scale, ranging from no education (1) to university degree (9). Intensity of exposure to Frisian was measured as the mean percentage of Frisian input the child received from his mother, father, siblings and other adults who looked after the child at least once per week. For each of these people the question had to be answered how often (s)he spoke Frisian to the child: ‘never’ (0%), ‘seldom’ (25%), ‘sometimes’ (50%), ‘usually’ (75%) and ‘always’ (100%). Intensity of exposure to Dutch at home was 100% minus intensity of exposure to Frisian at home. As SES and IQ (Rice and Hoffman, 2015) and exposure (Pearson et al., 1997) have been shown to be related to vocabulary learning we included these as control variables.

**Table 1:** Descriptive characteristics of the participants.

	Mean (*SD*) (*n* = 120)	Range	Maximum possible score
Age at time 1	70 (7)	59–83	
Age at time 2	82 (7)	71–95	
Age at time 3	94 (7)	83–107	
IQ	106 (15)	73–144	144
SES	6.9 (1.3)	3.5–9	9
% FR	63 (29)	0–100	100

*Age, age in months; IQ, intelligence quotient; SES, socioeconomic status; % FR, intensity of exposure to Frisian at home.*

### Measurement Instruments

#### Frisian Receptive Vocabulary

Frisian receptive vocabulary was measured with a task that was based on the Peabody Picture Vocabulary Test-III-NL (PPVT-III-NL; Schlichting, 2005), which is the Dutch version of the PPVT-III (Dunn and Dunn, 1997). Permission was obtained from the publisher to use this Frisian adaptation for research purposes. In this Frisian adaptation [see Bosma et al. (2016) for more details], only the first 144 words of the Dutch PPVT were used. These items suffice to test the vocabulary knowledge of the children in our age range. To make sure that all children completed all items, we did not use basal and ceiling criteria.

Words were assigned to four different cognate categories that differed with respect to degree of cross-language similarity: (1) identical cognates, such as Frisian *poes* [pus] and Dutch *poes* [pus] ‘cat, (2) non-identical cognates that exhibit a simple phonological regularity between Frisian and Dutch, such as *wâld* [wͻ:t] – *woud* [wʱut] ‘forest’, (3) non-identical cognates that do not exhibit a simple phonological regularity between Frisian and Dutch, such as Frisian *amer* [amər] and Dutch *emmer* [𝜀mər] ‘bucket’ and (4) non-cognates, such as Frisian *bern* [b𝜀:n] and Dutch *kind* [kɪnt] ‘child’.

Category (2) comprised items that exhibit a regularity of one, two or three phonemes. An overview of all cross-linguistic phonological regularities of category 2 and some examples can be found in **Table 2**. The vast majority of the items in category (3) were cognates without a cross-linguistic regularity (34 items). Two items followed a more complex cross-linguistic regularity that involves four phonemes. In order to check if the outcomes, in particular differences between category 2 and category 3, were affected by these two items, analyses were run both with and without these items.

**Table 2:** Cross-linguistic phonological regularities category 2.

Frisian phoneme(s)	Frisian example	Dutch phoneme(s)	Dutch example	English translation
[u:]	klûs [klu:s]	[œy]	kluis [klœys]	safe
[u]	pûlfrucht [pulfrøxt]	[ø 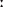 ]	peulvrucht [pø 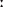 lvrøxt]	legume
	ûnder [undər]	[o]	onder [ondər]	under
[sk]	skep [sk𝜀p]	[sx]	schep [sx𝜀p]	shovel
[ͻ:n]	hân [hͻ:n]	[ʱnt]	hand [hʱnt]	hand
[ͻ:t]	kâld [kͻ:t]	[ʱut]	koud [kʱut]	cold
[a:]	daam [da:m]	[ʱ]	dam [dʱm]	dam
[ər]	ferstelber [fərst𝜀lbər]	[a:r]	verstelbaar [vərst𝜀lba:R]	adjustable
[I.ə]	easten [I.əstən]	[o:]	oosten [o:stən]	east
[i]	dolfyn [dolfin]	[𝜀i]	dolfijn [dolf𝜀in]	dolphin
[(k)jə]	timmerje [tImərjə]	[ən]	timmeren [tImərən]	to hammer
[j𝜀rjə]	dosearje [do:sj𝜀rjə]	[I.ərən]	doceren [do:sI.ərən]	to teach
[tsjə]	kadootsje [kado:tsjə]	[tjə]	cadeautje [kado:tjə]	(little) present
[kə]	groepke [grupkə]	[jə]	groepje [xrupjə]]	(small) group
	boeid [buit]	[xə]	geboeid [xəbuit]	chained

As a consequence of how we defined the cognate categories, there was a significant difference between the four categories regarding the number of phoneme differences between the Frisian and Dutch translation equivalents. *F*(3,140) = 93.47, *p* < 0.001, $\eta_{p}^{2}$ = 0.67 (category 1: *M* = 0.00, *SD* = 0.00; category 2: *M* = 1.86, *SD* = 0.99; category 3: *M* = 2.92, *SD* = 1.25; category 4: *M* = 5.72, *SD* = 2.50). Pairwise comparisons showed that all differences between categories were significant at the *p* < 0.01 level. There were, however, no significant differences between the four cognate categories with respect to the number of phonemes per word, *F*(3,140) = 0.95, *p* = 0.42, $\eta_{p}^{2}$ = 0.02 (category 1: *M* = 6.17, *SD* = 2.06; category 2: *M* = 6.75, *SD* = 2.63; category 3: *M* = 5.83, *SD* = 2.04; category 4: *M* = 6.17, *SD* = 2.60).

Furthermore, it was ensured that there were no word frequency differences between the four categories. The only available corpus for Frisian is a non-lemmatized database of standardized written language, which is not representative of the language that is spoken by speakers of Frisian (Breuker, 1993). Therefore, we used frequencies per million words from two Dutch corpora instead: CELEX (Center for Lexical Information, 1993), which is a corpus of written Dutch that was also used for the PPVT-III-NL, and *Corpus Gesproken Nederlands* (“Corpus Spoken Dutch”; CGN; Nederlandse Taalunie, 2004), which is a corpus of spoken Dutch. As Frisian and Dutch are closely related languages, the Dutch frequencies were thought to be representative of the Frisian frequencies. As frequency is perceived logarithmically, we calculated Zipf scores (Van Heuven et al., 2014), which are based on logarithmic (10-log) instead of absolute frequencies.

The four cognate categories each had about the same frequencies in CELEX and CGN, which was also confirmed by the high correlation between the CELEX and the CGN frequencies, *r* = 0.75, *p* < 0.001. A One-Way ANOVA with category as the independent variable and CELEX frequencies as the dependent variable showed that there was no significant effect of CELEX frequency, *F*(3,140) = 0.24, *p* = 0.87, and that the CELEX frequencies of category 1 (*M* = 3.82, *SD* = 0.92), category 2 (*M* = 3.85, *SD* = 1.39), category 3 (*M* = 4.04, *SD* = 1.22) and category 4 (*M* = 3.96, *SD* = 1.37) could be assumed to be the same. A One-Way ANOVA with category as the independent variable and CGN frequencies as the dependent variable showed that there was also no significant effect of CGN frequency, *F*(3,140) = 0.40, *p* = 0.76, and that the CGN frequencies of category 1 (*M* = 3.71, *SD* = 0.66), category 2 (*M* = 3.79, *SD* = 0.86), category 3 (*M* = 3.93, *SD* = 1.05) and category 4 (*M* = 3.85, *SD* = 0.99) could be assumed to be the same. Furthermore, Cronbach’s alpha, as calculated at time 1, showed that the internal consistency of the items in the test was sufficient, α = 0.76.

#### Verbal Memory

Both verbal short-term memory and verbal working memory were measured, as this allowed us to separate the storage component of verbal working memory from the processing component. Verbal short-term memory was measured with the Forward Digit Span and verbal working memory with the Backward Digit Span. These tasks were based on the Alloway Working Memory Assessment (AWMA; Alloway, 2012) and translated to Dutch. It was assumed that all children were able to count to 10 in Dutch, since Dutch is the main language of education and all children had spent at least 1 year in education at the first time of testing. In the forward version of the task, children had to repeat sequences of digits in the same order, whereas in the Backward Digit Span, they had to repeat them in reversed order. The Forward Digit Span is considered a measure of verbal short-term memory, because it only requires the storage of the digits. The Backward Digit Span, in contrast, is considered a measure of verbal working memory, because the added requirement to recall the digits in reversed order imposes a substantial processing load on the child (Alloway et al., 2008).

The task started with sequences of one digit, after which the sequences became increasingly longer. Per block, there were six trials and after three incorrect trials within one block the task stopped. When the child repeated the first four trials within one block correctly, he or she automatically continued with the next block and received a score of six. When the child repeated four out of the first five trials correctly, he or she also automatically continued with the next block and received a score of five. The AWMA procedure (Alloway, 2012) was applied for scoring. Trials were scored as incorrect if (part of) the sequence was incorrect, if children recalled one or more digits incorrectly, or if they omitted one or more digits. There were seven blocks for both the Forward and the Backward Digit Span, so the scores could range from 0 to 42.

### Procedure

The schools distributed consent forms and folders providing information about the experiment among the parents of the children. Children whose parents had signed the consent form were tested individually in a quiet room at school, except for one child at time 1, four children at time 2 and five children at time 3, who were tested at home. The children were tested by the first author and two research assistants, who all had a native level command of both Frisian and Dutch. The tasks in this study were part of a larger test battery that included language and cognitive tasks that are not reported on in the current study. Children were tested on all tasks at all three time points.

## Results

### Descriptive Statistics

Means and standard deviations for the Forward Digit Span, the Backward Digit Span and the four cognate categories are given in **Table 3**. Repeated measures ANOVAs showed that over time, children improved on all measures, *p* < 0.001. Bivariate correlations among all variables at time 1, 2 and 3 are reported in the **Appendix**.

**Table 3:** Means and standard deviations for the Forward Digit Span, the Backward Digit Span and the four cognate categories.

	Time 1 *M* (*SD*)	Time 2 *M* (*SD*)	Time 3 *M* (*SD*)	*p*	η^2^
**Memory measures**					
Forward Digit Span	20.11 (3.51)	22.47 (3.92)	24.11 (3.35)	<0.001	0.534
Backward Digit Span	12.75 (2.92)	14.90 (2.88)	16.47 (3.57)	<0.001	0.391
**Cognate categories**					
Category 1	23.11 (2.46)	25.18 (2.16)	26.23 (2.22)	<0.001	0.440
Category 2	22.35 (2.92)	24.42 (2.59)	26.23 (2.75)	<0.001	0.475
Category 3	22.79 (3.17)	24.51 (2.73)	25.99 (2.49)	<0.001	0.403
Category 4	22.03 (4.05)	23.87 (3.61)	24.78 (2.92)	<0.001	0.279

*Category 1 = identical cognates; category 2 = cognates with a simple rule; category 3 = cognates without a simple rule; category 4 = non-cognates.*

### Mixed Models Analysis

The research question of the current study was whether verbal working memory is related to the acquisition of cross-linguistic phonological regularities. We investigated this research question by examining whether the Backward Digit Span (verbal working memory) had a stronger effect on vocabulary items from cognate category (2) than on vocabulary items from cognate category (1), (3) and (4). In order to answer the research question we used a cumulative link mixed model. The mixed model was run, using the clmm function as implemented in the R package ordinal (Christensen, 2015). We entered Frisian receptive vocabulary accuracy as the ordered dependent variable, with 1 indicating a correct answer and 0 indicating an incorrect answer. We included random intercepts for subject and item, as both of these variables had repeated values. Including random intercepts would allow us to generalize the outcomes to the larger population of Frisian-Dutch bilingual children and to other items. A manual stepwise model selection procedure was carried out in which factors were added in such a way that the Akaike Information Criterion (AIC) was minimized. This procedure was applied with Category and Backward Digit Span as the main predictors of our study. In addition, the following predictors were added as control variables: Time, Frisian exposure at home, SES, IQ, Age and Forward Digit Span (verbal short-term memory). Time was added as an ordered factor, with 1 < 2 < 3. All of the predictors, except for Category, improved the model fit and were thus included in the final model. As expected, higher scores on exposure, SES, non-verbal IQ, age and Backward Digit Span were related to better performance on Frisian receptive vocabulary. Time was not a significant predictor, but was added to the final model, as the AIC showed that it did improve the fit. Furthermore, it must be noted that the Forward Digit Span was only significant when the Backward Digit Span was not included in the model.

**FIGURE 1:**
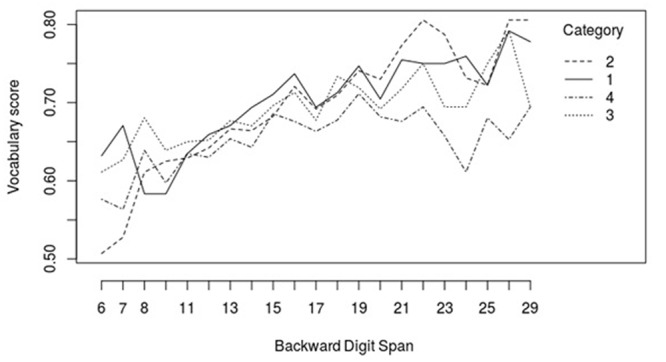
Interaction effect between Category and Backward Digit Span on Frisian receptive vocabulary accuracy. Category 1 = identical cognates; category 2 = cognates with a simple rule; category 3 = cognates without a simple rule; category 4 = non-cognates.

The model was further refined in an exploratory way by adding potential interactions between the predictors, including Category. This was done in order to increase the amount of explained variance, which would give a better focus on the variables of interest. Interactions between Category and Exposure, Category and Forward Digit Span, and Category and Backward Digit Span significantly improved the model fit and were therefore included in the final model. Models with three-way interactions including Time did not converge. In order to examine the interaction effects in more detail, the model was run four times with different reference levels for Category (1, 2, 3, and 4). We will first discuss the control interactions (Category × Exposure, Category × Forward Digit Span), followed by the interaction of interest (Category × Backward Digit Span). The interaction effect between Category and Exposure showed that the effect of Exposure on Frisian vocabulary was strongest for category (4), followed by category (3), category (2), and category (1) (4 > 3 > 2 > 1). The interaction effect between Category and Forward Digit Span showed that the effect of Forward Digit Span on Frisian vocabulary was significantly stronger for items from category (1) than for items from category (3) and (4), and stronger for items from category (2) than for items from category (4) (1 > 3, 4; 2 > 4). This shows that the effect of Forward Digit Span was stronger for items with a high degree of overlap across Frisian and Dutch than for items with a low degree of overlap, although the effect of Forward Digit Span on two adjacent categories was never significantly different. Finally, we examined the interaction effect between Category and Backward Digit Span, which was the focus of the current study. The results showed that the Backward Digit Span had a significantly stronger effect on vocabulary items from category (2) than on vocabulary items from category (1), (3) and (4). The differences between categories (1), (3) and (4) were statistically non-significant (2 > 1, 3, 4). The results of the final model are reported in **Table 4**, with category (2) as the reference level, as this category was the focus of our study. **Figure 1** shows the interaction effect between Category and Backward Digit Span. In this figure, it can be seen that the slope of category (2) is steeper than the slope of the other three categories.

**Table 4:** Fixed effects from the final model with Frisian receptive vocabulary accuracy as dependent variable and category 2 as reference level.

Effect	Estimate	Std. Error	*Z*-value	*P*-value
Time.Linear	0.081	0.073	1.118	0.264
Time.Quadratic	–0.043	0.022	–1.941	0.052
Category 1	0.316	0.610	0.517	0.605
Category 3	–0.210	0.605	–0.347	0.729
Category 4	–0.520	0.605	–0.859	0.390
Frisian exposure	0.079	0.035	2.279	0.023*
SES	0.100	0.026	3.778	<0.001***
Non-verbal IQ	0.085	0.028	3.056	0.002**
Age	0.280	0.049	5.730	<0.001***
Forward Digit Span	0.023	0.036	0.650	0.515424
Backward Digit Span	0.157	0.034	4.641	<0.001***
Category 1 × exposure	–0.123	0.037	–3.314	<0.001***
Category 3 × exposure	0.212	0.036	5.963	<0.001***
Category 4 × exposure	0.368	0.035	10.484	<0.001***
Category 1 × Forward Digit Span	0.066	0.044	1.490	0.136195
Category 3 × Forward Digit Span	–0.032	0.042	–0.768	0.442396
Category 4 × Forward Digit Span	–0.087	0.041	–2.108	0.035*
Category 1 × Backward Digit Span	–0.124	0.045	–2.785	0.005**
Category 3 × Backward Digit Span	–0.151	0.042	–3.563	<0.001***
Category 4 × Backward Digit Span	–0.157	0.042	–3.758	<0.001***

*Category 1 = identical cognates; category 2 = cognates with a simple rule; category 3 = cognates without a simple rule; category 4 = non-cognates; ^∗^*p* < 0.05; ^∗∗^*p* < 0.01; ^∗∗∗^*p* < 0.001.*

As explained in the Method section, there were two items from category (3) that followed a more complex cross-linguistic phonological regularity. In order to check if these items affected the outcomes, the analyses described above were rerun without these items. The results showed that excluding these two items did not affect the outcomes.

We considered that the effect of Time, Exposure, SES, IQ, Age, Forward Digit Span, Backward Digit Span and Category may be different per subject and per item. Therefore, we added several combinations of these variables as random slopes to subject and item. We found that adding Age as random slope to subject and the factors Age, Time and Forward Digit Span as random slopes to item improved the model fit and slightly changed the results, with Time now being a significant predictor, *p* = 0.016. However, when we tried to rerun this model with the same random slopes but without the Backward Digit Span as a predictor, the model did not converge. The same problem occurred when we tried to rerun the model with the same random slopes but without the two items from category (3) that followed a more complex cross-linguistic phonological regularity.

## Discussion

Previous research has shown that verbal working memory is related to the acquisition of grammar, but not vocabulary (e.g., Engel de Abreu and Gathercole, 2012; Verhagen and Leseman, 2016). This suggests that verbal working memory supports the acquisition of linguistic regularities. In the present study, we investigated this hypothesis by examining whether verbal working memory is also related to the acquisition of cross-linguistic phonological regularities, such as Frisian *-âld* [ͻ:t] and Dutch *-oud* [ʱut], as in the cognate pairs *kâld* [kͻ:t] – *koud* [kʱut] ‘cold’ and *wâld* [wͻ:t] – *woud* [wʱut] ‘forest’. In order to answer this question, 5- to 8-year-old Frisian-Dutch bilingual children were tested annually for a 3-year period on verbal working memory and a Frisian receptive vocabulary task with four cognate categories: (1) identical cognates, (2) non-identical cognates that either do or (3) do not exhibit a phonological regularity between Frisian and Dutch, and (4) non-cognates. As age, non-verbal IQ (Rice and Hoffman, 2015), exposure (Pearson et al., 1997), SES (Rice and Hoffman, 2015) and verbal short-term memory (Engel de Abreu and Gathercole, 2012) have previously been shown to be related to vocabulary acquisition, these were also measured and included as control variables.

In line with previous studies, the results showed significant main effects of age, SES, non-verbal IQ and exposure on Frisian receptive vocabulary, with higher scores on these variables resulting in better vocabulary scores. Verbal short-term memory was only significant when verbal working memory was not included in the model. When a model was run that included both verbal short-term memory and verbal working memory, only verbal working memory came out as a significant predictor. This is probably due to the fact that, according to some definitions (Baddeley and Hitch, 1974; Baddeley, 1986), verbal short-term memory is part of verbal working memory. In addition to these main effects, we found interaction effects between cognate category and exposure, cognate category and verbal short-term memory, and cognate category and verbal working memory. As the first two interactions were only added as control variables to improve the model, we will not discuss these here, but instead concentrate on the interaction between cognate category and verbal working memory, which was the focus of the current study. The interaction between cognate category and verbal working memory showed that verbal working memory had a significantly stronger effect on cognate category (2) than on cognate category (1), (3) and (4). This suggests that verbal working memory supports the acquisition of regularities across the Frisian and Dutch phonological systems.

The finding that verbal working memory supports the acquisition of cross-linguistic phonological regularities is noteworthy for the following reasons. First, it provides psycholinguistic evidence for the existence of cross-linguistic phonological regularities (Sjölin, 1976; Rys, 2009; Taeldeman, 2013). Second, it confirms that bilingual children learn these regularities (Bosma et al., 2016) by showing that they do so on the basis of a general cognitive capacity, namely verbal working memory. Third, the results suggest that the acquisition of phonological regularities across languages shares important characteristics with the acquisition of grammatical rules within a language, which has previously been shown to be related to verbal working memory (Gottardo et al., 1996; McDonald, 2008; Engel de Abreu and Gathercole, 2012; Verhagen and Leseman, 2016). Fourth, as both the acquisition of grammar and the acquisition of cross-linguistic phonological regularities are related to verbal working memory, this suggests that verbal working memory plays a role in the acquisition of linguistic regularities.

These results can well be explained within the framework of Bybee’s (1995, 2001, 2008, 2010) network model, although we do not exclude the possibility that other models may also fit the data. As Costa et al. (2005) already mentioned, the spreading of activation within the bilingual lexicon (Dijkstra et al., 2010; Bosma et al., 2016) is similar to the spreading of activation within the monolingual lexicon (Gonnerman et al., 2007). Within the network model, this implies that related words are stored together, regardless of whether they belong to the same or to a different language. This suggests that the acquisition of phonological regularities across languages shares important characteristics with the acquisition of grammatical relations within a language, as they are both generalizations that emerge from schemas of phonologically and semantically related words. Our finding that the acquisition of cross-linguistic phonological regularities is related to verbal working memory supports this suggestion, as previous research has shown that the acquisition of grammar is also related to verbal working memory (Gottardo et al., 1996; McDonald, 2008; Engel de Abreu and Gathercole, 2012; Verhagen and Leseman, 2016). In terms of Bybee’s network model, this parallel between cross-linguistic regularities and grammar suggests that verbal working memory plays a role in the formation of linguistic schemas through categorization and/or their productive use through analogy, a view that is in line with previous evidence that verbal working memory also plays a role in the categorization of non-linguistic items (Lewandowsky, 2011; Lewandowsky et al., 2012) and in non-linguistic analogical reasoning (Waltz et al., 2000).

There are a number of limitations to the present study that are relevant to mention. First, although we only investigated the role of verbal working memory in the acquisition of cross-linguistic phonological regularities, other cognitive skills might play a role as well. An example of another skill that may influence the acquisition of cross-linguistic phonological regularities is phonological awareness, which is the conscious ability to detect and differentiate between the sounds of a word and to manipulate phonemes to create new words. Previous research has shown that phonological awareness positively influences reading and spelling acquisition, because children with high phonological awareness skills are better able to identify and use letter-sound correspondences (Ehri et al., 2001). In the same way, phonological awareness might help children to identify and use correspondences between the phonological systems of two languages.

A second limitation of the current study is that we investigated the acquisition of cross-linguistic phonological regularities in general, without zooming in on differences that might exist between different types of regularities. Within the network model, it is argued that the productivity of a regularity is to a large extent determined by its type frequency, that is, the number of items that follow that regularity. The more items a schema encompasses, the stronger it is, and the higher the likelihood that the pattern will be extended to novel items. Type frequency interacts with degree of schematicity, that is, the degree of dissimilarity of the members of a class. Highly schematic classes include a wide range of dissimilar items. For example, the English past tense has a high degree of schematicity, as it can be applied to all verbs, no matter their phonological form. In the network model, it is argued that a high type frequency in combination with a high degree of schematicity results in a maximally productive construction. For future research, it would be interesting to examine to what extent the acquisition of cross-linguistic phonological regularities depends on type frequency and degree of schematicity and whether type frequency and schematicity interact with verbal working memory.

Taken together, the main finding of this study is that verbal working memory is related to the acquisition of cross-linguistic phonological regularities. This supports the hypothesis that verbal working memory plays a role in the acquisition of linguistic regularities, thus providing more insight into the mechanisms that facilitate language acquisition.

## Ethics Statement

All the parents of the participating children gave their written informed consent, as was stated in the Section “Materials and Methods” of our paper. Unfortunately, the study was not officially evaluated by an ethics committee before the start of the study due to a miscommunication. In hindsight, the ethics committee of the University of Amsterdam evaluated the information folder and the informed consent form that we used and came to the conclusion that the research had been conducted with the wellbeing of the participants in mind.

## Author Contributions

All authors listed have made a substantial, direct and intellectual contribution to the work, and approved it for publication.

## Conflict of Interest Statement

The authors declare that the research was conducted in the absence of any commercial or financial relationships that could be construed as a potential conflict of interest.

## APPENDIX

**Table:** Bivariate correlations among all variables at Time 1.

	SES	IQ	% FR	FW DS	BW DS	cat1	cat2	cat3	cat4
Age	–0.104	–0.013	0.095	0.259**	0.265**	0.341***	0.405***	0.178*	0.271**
SES	–	0.062	–0.253**	0.145	0.047	0.092	–0.052	0.035	–0.107
IQ		–	–0.014	0.208*	0.374***	0.265**	0.371***	0.072	0.149
% FR			–	0.002	0.039	–0.034	0.244**	0.495***	0.625***
FW DS				–	0.479***	0.304***	0.265*	0.098	0.112
BW DS					–	0.245*	0.422***	0.080	0.160
cat1						–	0.391***	0.171	0.233**
cat2							–	0.359***	0.493***
cat3								–	0.604***

*Cat1 = identical cognates; cat2 = cognates with a simple rule; cat3 = cognates without a simple rule; cat4 = non-cognates; % FR = intensity of exposure to Frisian at home; FW DS, Forward Digit Span; BW DS, Backward Digit Span; ^∗^*p* ≤ 0.05; ^∗∗^*p* ≤ 0.01; ^∗∗∗^*p* ≤ 0.001.*

**Table:** Bivariate correlations among all variables at Time 2.

	SES	IQ	% FR	FW DS	BW DS	cat1	cat2	cat3	cat4
Age	–0.115	–0.026	0.100	0.074	0.126	0.301***	0.400***	0.330***	0.159
SES	–	0.039	–0.244**	0.129	0.141	0.089	0.184*	0.057	–0.118
IQ		–	–0.007	0.209*	0.265**	–0.080	0.153	0.122	0.047
% FR			–	–0.012	0.016	–0.124	0.195*	0.412***	0.620***
FW DS				–	0.491***	0.158	0.154	0.059	–0.045
BW DS					–	0.116	0.284**	0.181*	0.122
cat1						–	0.348***	0.176	0.136
cat2							–	0.526***	0.400***
cat3								–	0.544***

*Cat1 = identical cognates; cat2 = cognates with a simple rule; cat3 = cognates without a simple rule; cat4 = non-cognates; % FR = intensity of exposure to Frisian at home; FW DS, Forward Digit Span; BW DS, Backward Digit Span; ^∗^*p* ≤ 0.05; ^∗∗^*p* ≤ 0.01; ^∗∗∗^*p* ≤ 0.001.*

**Table:** Bivariate correlations among all variables at Time 3.

	SES	IQ	% FR	FW DS	BW DS	cat1	cat2	cat3	cat4
age	–0.123	–0.028	0.099	0.124	0.102	0.277**	0.251**	0.150	0.237**
SES	–	0.039	–0.244**	0.173	0.151	0.071	0.251**	0.115	0.016
IQ		–	–0.007	0.171	0.332***	0.214*	0.249**	0.184*	0.086
% FR			–	–0.039	–0.001	–0.118	–0.097	0.455***	0.585***
FW DS				–	0.380***	0.280**	0.268**	0.074	–0.052
BW DS					–	0.292***	0.383***	0.147	0.016
cat1						–	0.588***	0.188*	0.211*
cat2							–	0.340***	0.229*
cat3								–	0.543***

*Cat1 = identical cognates; cat2 = cognates with a simple rule; cat3 = cognates without a simple rule; cat4 = non-cognates; % FR = intensity of exposure to Frisian at home; FW DS, Forward Digit Span; BW DS, Backward Digit Span; ^∗^*p* < 0.05; ^∗∗^*p* < 0.01; ^∗∗∗^*p* < 0.001.*
